# Cross-disorder genetic analysis of immune diseases reveals distinct gene associations that converge on common pathways

**DOI:** 10.1038/s41467-023-38389-6

**Published:** 2023-05-12

**Authors:** Pietro Demela, Nicola Pirastu, Blagoje Soskic

**Affiliations:** Human Technopole, Viale Rita Levi-Montalcini 1, 20157 Milan, Italy

**Keywords:** Immunogenetics, Autoimmunity, Genome-wide association studies

## Abstract

Genome-wide association studies (GWAS) have mapped thousands of susceptibility loci associated with immune-mediated diseases. To assess the extent of the genetic sharing across nine immune-mediated diseases we apply genomic structural equation modelling to GWAS data from European populations. We identify three disease groups: gastrointestinal tract diseases, rheumatic and systemic diseases, and allergic diseases. Although loci associated with the disease groups are highly specific, they converge on perturbing the same pathways. Finally, we test for colocalization between loci and single-cell eQTLs derived from peripheral blood mononuclear cells. We identify the causal route by which 46 loci predispose to three disease groups and find evidence for eight genes being candidates for drug repurposing. Taken together, here we show that different constellations of diseases have distinct patterns of genetic associations, but that associated loci converge on perturbing different nodes in T cell activation and signalling pathways.

## Abstract (web summary)

Immune related diseases have been found to have overlapping genetic susceptibility loci. Here the authors perform cross-disorder genetic analysis to uncover three genetic groups of immune diseases that converge on the same immune cells and pathways.

## Introduction

Immune-mediated diseases are chronic and disabling conditions where the immune system attacks healthy tissue, leading to its destruction. It is well documented that these diseases co-occur within families and that multiple immune diseases are likely to occur in the same individual^1–3^ suggesting that immune diseases have a shared genetic basis.

Genome-wide association studies (GWAS) have identified thousands of susceptibility loci associated with immune-mediated diseases, many of which have been observed in multiple diseases^4,5^. For example, the major histocompatibility complex locus is associated with most autoimmune diseases^6^. Another example is a locus containing *CTLA4* which is associated with multiple immune diseases including rheumatoid arthritis (RA), coeliac disease (CeD), type 1 diabetes (T1D) and Hashimoto thyroiditis (Ht)^7–10^. Targeting the CTLA-4 pathway has been successful in tumour immunotherapy, however in more than 60% of patients, CTLA-4 blockade leads to multiorgan autoimmune reaction^11^. In contrast, the property of CTLA-4 to bind the costimulatory molecules is extensively used as a treatment for RA^12^.

Understanding the pleiotropy of genetic associations is critical, as it can reveal common disease mechanisms and pathogenic pathways. A cross-disorder genomic analysis could identify shared mechanisms and potential targets for drug repurposing. By combining cases and controls across immune diseases, recent work identified 224 shared associations, improved fine-mapping, and revealed shared disease genes such as *RGS1*^13^. Similarly, a study using local genetic correlation showed widespread sharing across traits^14^. For example, T1D and Systemic Lupus Erythematosus (SLE) shared 18 loci. Another study assessed the regulatory activity of immune disease-associated SNPs and showed that shared genes were highly connected and were involved in immune pathways^15^. Although it has been established that immune phenotypes have a shared genetic predisposition, further detailed and systematic analysis is necessary to understand the causes and structure of such sharing. In particular, it is unclear whether sharing is equally distributed across immune diseases (i.e. is there a common factor conferring general risk for all immune diseases?) or whether there are subgroups of immune diseases that are more similar to each other than the rest.

In this work, we sought to investigate common factors representing general risk across immune-mediated diseases. To examine the genetic architecture of nine immune-mediated diseases we applied genomic structural equation modelling (genomic SEM)^16^ to GWAS data. This revealed three groups of diseases: the first consisted of diseases affecting the gastrointestinal tract, the second consisted of rheumatic and systemic disorders and the third group represented allergic diseases. Each group had unique genetic architecture and only a limited number of loci were in common among the groups. Collectively, our results provide new insights into shared mechanisms of genetic risk for immune-mediated diseases and prioritise drug targets that could be used for multiple immune disorders.

## Results

### Factor analysis reveals three groups of immune diseases

**Fig. 1:**
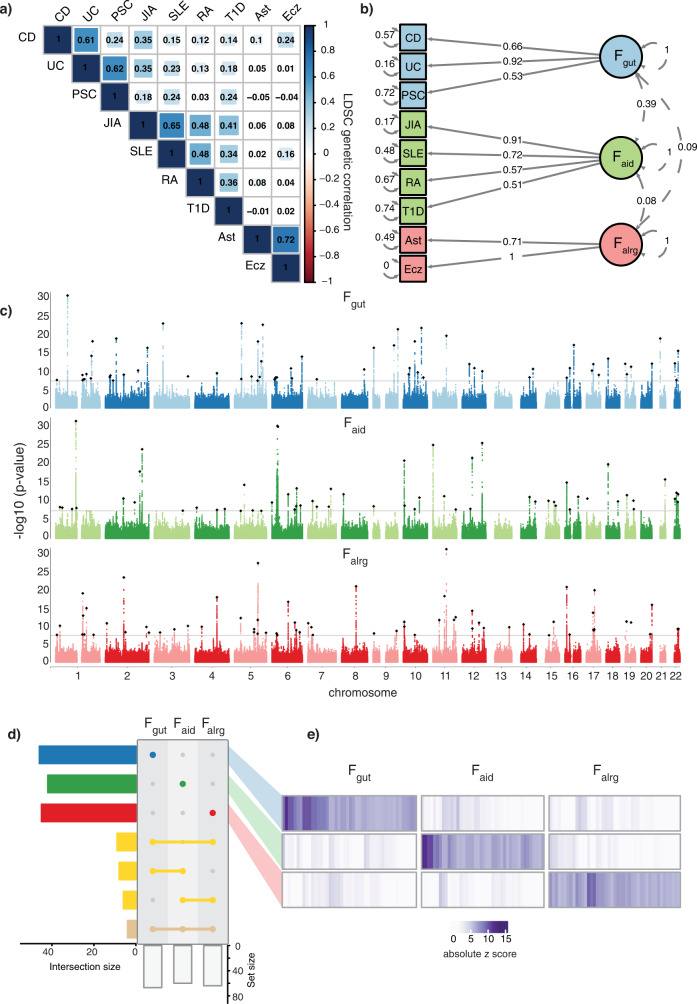
Three groups of immune-mediated diseases have distinct patterns of genetic associations. **a** Genetic correlation matrix of nine immune-mediated diseases estimated with LD score regression. Shades of blue and red indicate positive and negative correlations respectively. Blue represents F_gut_, green F_aid_ and red F_alrg_. **b** Path diagram of the three-factor model of immune-mediated diseases. Colours represent different factors. Latent variables representing common genetic factors are depicted as circles. Standardised loadings (one-headed arrows), residual variances (two-headed arrows connecting the variable with itself) and covariances (two-headed arrows connecting latent variables) are shown. **c** Manhattan plots of SNP-specific effects on each factor. Black rhomboids represent lead SNPs and a solid line indicates the genome-wide significant threshold (*p* value = 5 × 10^−8^). Genomic SEM (WLS estimation method) was used to conduct the factor GWAS. **d** UpSet plot showing the overlap between significant genomic regions associated with different factors; intersection size indicates the number of overlapping regions. Asymmetric overlaps (e.g. two regions in one factor overlapping with one region in the other) are counted as one overlap. Yellow represents overlapping genomic regions. **e** Heatmap of absolute z-scores of factor-specific genomic regions. Each column corresponds to a lead SNP, with rows corresponding to factors. Hierarchical clustering was applied to the columns, with breaks along columns separating the factor-specific lead SNPs. CD Crohn’s disease, UC ulcerative colitis, PSC primary sclerosing cholangitis, JIA juvenile idiopathic arthritis, SLE systemic lupus erythematosus, RA rheumatoid arthritis, T1D type 1 diabetes, Ecz eczema, Ast asthma.

To investigate whether there is a common genetic factor underlying multiple immune-mediated diseases, we first used the multivariate LD score regression implementation in genomic SEM^16,17^ to estimate genetic correlations among nine diseases (Crohn’s disease, CD; ulcerative colitis, UC; primary sclerosing cholangitis, PSC; juvenile idiopathic arthritis, JIA; systemic lupus erythematosus, SLE; rheumatoid arthritis, RA; type 1 diabetes, T1D; eczema, Ecz; asthma, Ast) (Fig. 1a, Supplementary Data 1). We collected GWAS summary statistics from European populations, and we selected studies that used genome-wide genotyping arrays, as it is required for accurate estimation of LD score regression. We observed three distinct groups of immune-mediated diseases that clustered together in the genetic correlation matrix (genetic correlation ≥0.4; group 1: CD, UC and PSC; group 2: RA, SLE, JIA and T1D; group 3: Ast and Ecz) (Supplementary Fig. 1a, Supplementary Data 2). To uncover the latent factors which represent shared variance components across diseases, we modelled the genetic variance-covariance matrices across traits using genomic SEM (Fig. 1b)^16^. By using the combination of SRMR and CFI estimates (see Methods), we were able to show that the genetic correlation structure was well described by a model using three factors (Supplementary Fig. 2a–e). Factor one consisted of diseases affecting the gastrointestinal tract (CD, UC and PSC). Factor two contained autoimmune diseases, which were largely rheumatic and systemic disorders (RA, SLE, JIA and T1D). Finally, factor three contained allergic diseases (Ast and Ecz) (Fig. 1b). Therefore, we refer to these factors as F_gut_, F_aid_ and F_alrg_, respectively.

To elucidate how genetic variation impacts the identified latent factors, we tested the association between common SNPs across GWAS studies and each of the latent factors. We discovered 194 genome-wide significant regions that are associated with latent factors, 67 for F_gut_, 60 for F_aid_ and 67 for F_alrg_ (Fig. 1c). Strikingly, the overlap between regions was modest, with only 30 out of 194 genomic regions overlapping among at least two factors, and only four regions overlapping across all three factors (Fig. 1d). The comparison of z-scores showed that this modest overlap was not due to  *p* value thresholding (i.e. the same region in another factor having a *p* value just below the threshold) (Fig. 1e). In addition, eosinophil counts^18^ showed the highest correlation with F_alrg_, giving further support to our factor definition (Supplementary Fig. 3a), and we did not observe a strong genetic correlation with lymphocyte or monocyte counts^18^ (Supplementary Fig. 3a).

Finally, we investigated whether the SNPs were acting via each of the three factors according to the proposed causal model or, whether SNPs had independent effects on the diseases that the factors are composed of. To do so, we computed the Q_SNP_ heterogeneity statistics (Methods). In short, Q_SNP_ allows us to identify SNPs that plausibly do not affect individual diseases exclusively by their associations with the latent common factors^16^. In other words, if the Q_SNP_ heterogeneity statistic is significant, it implies that the tested SNP acts at least partially independently of the latent factors. Our results show that only 9% of loci were significant for Q_SNP_ heterogeneity (18/194) (Supplementary Fig. 4a), suggesting that the three-factor model explained the genetic structure at the individual SNP level for 90% of identified regions.

### Latent factors have a distinct genetic architecture

**Fig. 2:**
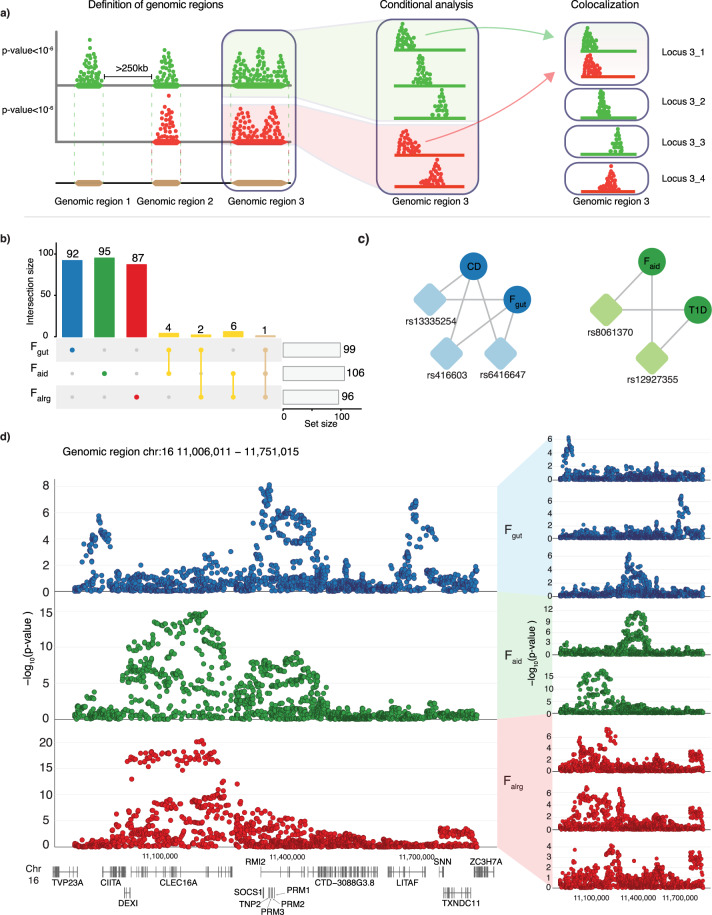
Latent factors have a distinct genetic architecture. **a** Schematic representation of the conditional analysis and colocalization strategy (see Methods). Colours represent different traits. **b** Blue, green and red represent loci that were specific for F_gut_, F_aid_ and F_alrg_, respectively, while yellow represents loci that are shared between factors. **c** Colocalization relationship between latent factors and traits in the region 16:11,006,011 − 11,751,015. Colours represent disease groups. Circles represent latent factors or traits, rsID of the lead SNP and rhomboids represent the loci that colocalize among traits. **d** Conditional analysis of the genomic region chr16:11,006,011−11,751,015. Locus-zoom plots of three different factors (blue for F_gut_, green for F_aid_, and red for F_alrg_) and the conditional loci for each of the latent factors in the regions are shown. Genomic SEM (WLS estimation method) was used to conduct the GWAS and COJO to estimate the conditional *p* values. CD Crohn’s disease, T1D type 1 diabetes.

An overlap of GWAS regions across two traits does not imply that the underlying causal mechanism is the same across traits. Given that many GWAS regions are complex and may contain multiple independent signals, we performed a systematic analysis of identified regions by combining conditional analysis with colocalization. Briefly, to increase the robustness of colocalization, we devised a statistical approach where the association signal is first decomposed into its conditionally independent components. Next, each component was used for colocalization testing allowing us to group similar association signals (Fig. 2a). This approach enabled resolving complex regions and discovering colocalization events for secondary signals, which would not have been possible by colocalizing the whole region.

Due to the challenges of the HLA region, we removed genomic regions encompassing *HLA* genes. We identified 301 independent signals (Supplementary Data 3, 4). Out of these 301 loci, 92 were specifically associated with F_gut_, 95 with F_aid_ and 87 with F_alrg_ (Supplementary Data 4, 5 and Fig. 2b). Only 12 loci were shared across any two factors, and only one was shared across all 3 factors. This further demonstrated that each group of diseases had a specific pattern of genetic associations. For example, a region on chromosome 16 encompassing multiple genes (11,006,011−11,751,015) had significant associations with all three factors (Fig. 2c, d). However, the conditional analysis and colocalization demonstrated that these signals were independent and not shared across factors. In this region, we identified three independent signals that colocalize between CD and F_gut_: rs12922863 (the closest gene *CIITA* which is involved in antigen presentation), rs416603 (the closest gene *TNP2* involved in the regulation of protein processing) and rs13335254 (the closest gene *LITAF* which regulates TNF-alpha expression). Similarly, F_aid_ had two independent signals which colocalized with T1D. The locus that was shared across all three groups of diseases is located on chromosome 4 (122,903,441–124,264,377) and encompasses a potent regulator of T and B cell proliferation *IL21*.

Taken together, we identified independent signals between factors and determined how each of the factors relate to individual diseases and their likely causal genes.

### Associated loci affect T-cell activation and signalling

**Fig. 3:**
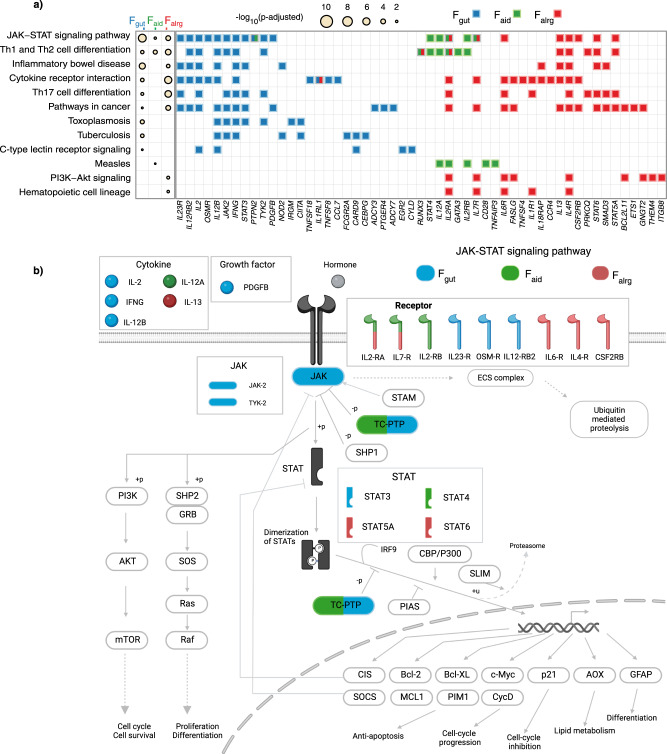
Factor-associated loci perturb different nodes of the same pathways. **a** KEGG pathway enrichment analysis of factor-associated genes. The heatmap shows KEGG pathways that were significantly enriched (*p* adjusted < 0.05) in factor-associated genes. The radius of the circle is proportional to the −log_10_(p-adjusted). *P* values were calculated with the hypergeometric test and corrected for multiple testing with the gprofiler-g:SCS. The tile plot shows enriched genes in each of the pathways. Blue, green and red represent the genes that contributed to the enrichment of F_gut_, F_aid_ and F_alrg_ respectively. **b** Schematic representation of JAK-STAT signalling pathway. Blue, green and red represent components of the pathway that contribute to the enrichment from F_gut_, F_aid_ and F_alrg_ respectively. Adapted from ‘Cytokine Signaling through the JAK-STAT Pathway’, by BioRender.com (2023). Retrieved from https://app.biorender.com/biorender-templates.

Identifying transdiagnostic risk pathways can uncover critical cell functions whose perturbations lead to immune system dysfunction and diseases. Therefore, we sought to translate factor-associated variants to cellular functions. Briefly, we queried the Open Targets Platform^19^, and for each lead SNP we retrieved the top prioritised gene based on the Variant-to-Gene (V2G) score. To test whether these genes are enriched in specific pathways, we performed pathway enrichment with gProfiler2 (Methods). This showed that the factor-associated genes were enriched in cytokine signalling, differentiation of T helper cells, immune diseases and response to pathogens (Fig. 3a and Supplementary Data 6). Given the modest overlap of factor-associated loci, we expected that the enriched pathways would be distinct across factors. However, factor-associated genes were largely enriched in the same pathways, although different genes were driving a pathway enrichment (Fig. 3a). For example, we observed that F_gut_, F_aid_ and F_alrg_ factor-associated loci were enriched in the JAK-STAT signalling pathway, which is critical for response to many cytokines (Fig. 3b). Nevertheless, the genes implicated in the JAK-STAT signalling pathway were largely distinct between factors, with only three genes shared between any pair of factors. Notably, the transcription factor *STAT3* was specifically associated with F_gut_, while *STAT4* was associated with F_aid_, and *STAT5A* and *STAT6* were associated with F_alrg_. This suggests that although trans-diagnostic risk loci are different for three groups of diseases, they converge on perturbing similar cellular functions.

**Fig. 4:**
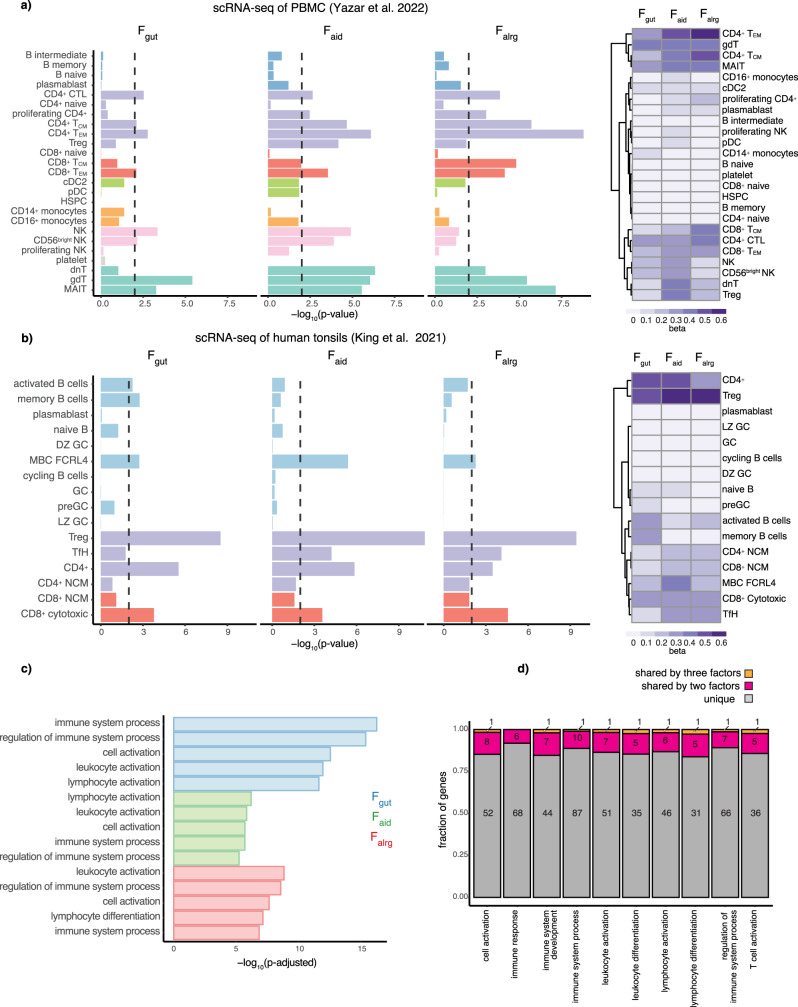
Factor-associated loci converge on T cells. MAGMA gene-property results of Onek1k PBMC dataset (**a**) and tonsillar cells (**b**). The barplot shows −log_10_(*p* value) of the enrichment. *P* values were estimated using MAGMA, using a one-sided test. Colours in the barplot represent groups of cells belonging to the same cell type. The heatmap shows regression coefficients from the MAGMA model. **c** The bar plot shows the −log_10_(p-adjusted) of the top five GO terms enriched in factor-associated genes. *P* values were calculated with the hypergeometric test and corrected for multiple testing with the gprofiler-g:SCS. Blue, green and red represent the GO terms for F_gut_, F_aid_ and F_alrg_ respectively. **d** The stacked-bar plot shows the number of genes unique or shared by the latent factors in the top 10 shared enriched GO terms. Grey represents genes unique to one of the factors, purple represents genes that are associated with two factors and orange represents genes that are associated with all three latent factors.

To test whether transdiagnostic risk variants also converge on a specific cell type, we conducted a MAGMA gene-property analysis implemented in CELLECT^20,21^. To do that we first used the OneK1K cohort^22^, which to date is the largest study containing single-cell RNA sequencing (scRNA-seq) data from 982 donors and 1.27 million peripheral blood mononuclear cells (PMBCs). We showed that there is an enrichment of F_gut_, F_aid_, and F_alrg_-associated loci in memory CD4^+^, CD8^+^ and unconventional T cells in all three disease groups (Fig. 4a). In contrast, we did not observe an enrichment of GWAS loci in naive T cells or B cell populations. Interestingly, NK cells were also enriched, but only for the F_gut_ and F_aid_ group of diseases. A similar pattern of enrichment was observed using S-LDSC (Supplementary Fig. 5a). In addition, given that tonsils are the secondary lymphoid organs where immune activation occurs, we verified T-cell enrichments using a study which profiled human tonsils at the single-cell level^23^. These data showed the same pattern of trans-diagnostic enrichment, observed in CD4 and CD8 T cells, with the strongest enrichment being observed in regulatory T cells (Fig. 4b). As observed in PBMC data, disease loci were generally not enriched in B cells. The exception to that was memory B cells expressing Fc receptor–like-4 (FCRL4 + B cells). FCRL4 + B cells are thought to be tissue-resident cells and have been identified as a potential target in RA therapy^24^, hence our results provide further genetic support for their modulation. Furthermore, we observed that disease loci were enriched in immune cells from gut^25^ and lung^26^ cell atlases, with the strongest enrichment observed in T cells as previously shown (Supplementary Fig. 6a, b). Nevertheless, we did not observe enrichment in epithelial or other non-immune cells. This shows that the cross-disease factors capture true immune signals that are shared across diseases.

Finally, we observed a similar enrichment pattern in biological processes across all three groups of diseases. Notably, genes in factor-associated loci were enriched for lymphocyte and immune activation (Fig. 4c and Supplementary Data 7), albeit this enrichment was driven by a distinct group of genes (Fig. 4d) as demonstrated previously.

Taken together, our data suggest that different groups of diseases have distinct patterns of genetic associations but that associated loci converge on perturbing different nodes in lymphocyte activation and cytokine signalling.

### Colocalization at factor loci identifies potential drug targets

**Fig. 5:**
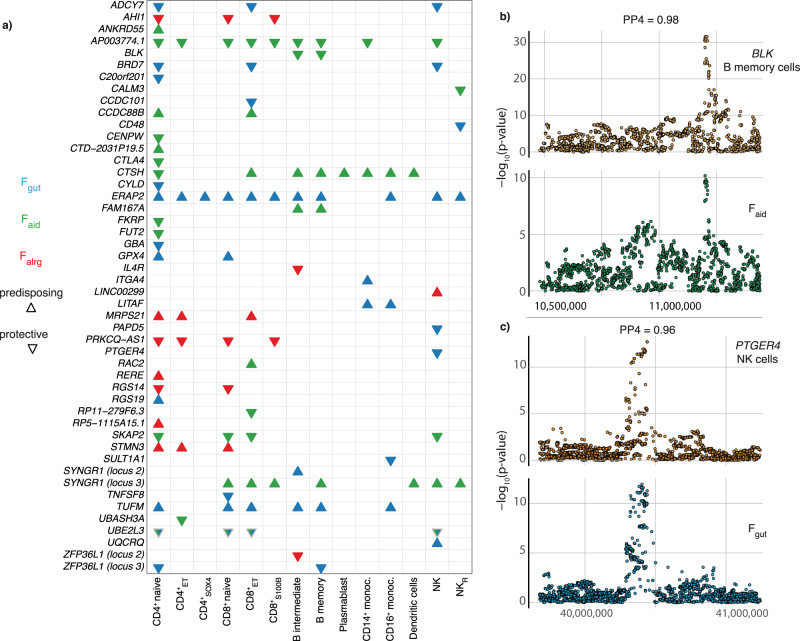
Colocalization of immune cell eQTLs prioritises cross-disease causal genes and identifies potential drug targets. **a** Colocalization and Mendelian Randomization results (see Methods) of eQTL predicting risk to the latent factors. Triangles pointing upwards indicate that an increase in gene expression increases disease risk, while triangles pointing downwards indicate a decrease in disease risk. Blue, green and red represent F_gut_, F_aid_ and F_alrg_ respectively. Only significant Mendelian Randomization results (p-value < 0.05) are shown. **b**–**c** Colocalization plots of latent factors and eQTLs. The posterior probability of colocalization (H4) is shown. **b** Locus-zoom plot representing the colocalization between the *BLK* gene in B memory cells and F_aid_. P-values refer to the SNP p-values derived from the factor GWAS and from the e-QTL dataset. **c** Locus-zoom plot representing the colocalization between the *PTGER4* gene in NK cells and F_gut_. *P* values refer to the SNP p-values derived from the factor GWAS and from the e-QTL dataset.

To assess whether variants associated with each disease group modulate gene expression in immune cells, we tested for colocalization between factor-associated loci and single-cell eQTLs (sc-eQTLs) derived from PMBCs from the OneK1K cohort^22^. Briefly, to identify independent and secondary eQTL signals we performed locus decomposition (see Methods) and colocalized with factor-associated loci using the Bayesian framework *coloc*^27^. We identified 46 colocalizations in F_gut_, 49 in F_aid_ and 20 in F_alrg_ with PP4 ≥ 0.9 (Supplementary Data 8). Finally, to determine whether an increase in gene expression predicts increased disease risk, we used Mendelian Randomization (MR) using the Wald ratio method (Fig. 5a and Supplementary Data 9). For example, an eQTL for Src family tyrosine kinase *BLK* present in naive memory B cells specifically colocalized with an association with the F_aid_ group of traits (Fig. 5b), with an increase in *BLK* expression associated with lower disease risk. This is consistent with the fact that rare variants that reduce BLK function have been demonstrated to induce SLE^28^. In another example, we observed that a locus associated with F_gut_ modulates the expression of Prostaglandin E Receptor 4 *PTGER4* (Fig. 5c). In this case, an increase in gene expression protects against the F_gut_ group of diseases.

One of the major hurdles of human genetics has been translating genetic findings into clinical insights. To identify potential drug targets, we used the Open Targets Platform^29^ and investigated whether colocalizing genes are known drug targets (Table 1). Of the 46 eQTL genes, eight are targeted by drugs which are either already used in the clinics or are in clinical trials. Four of these eight have been previously used in autoimmune diseases, while the other four represent potential candidates for drug repurposing. For example, our data shows that the increase in expression of a key immune regulator *CTLA4* is protective against the F_aid_ group of diseases. The property of CTLA-4 to regulate the immune system has long been exploited in the treatment of RA^12^. Similarly, an inhibitor for Integrin Subunit Alpha 4 *ITGA4* has been trialled in UC and CD (Open Targets database and Table 1). Our data gives further genetic evidence that an increase in *ITGA4* expression leads to an increased risk for F_gut_ diseases, and therefore it is plausible that inhibiting *ITGA4* would be beneficial not only in CD and UC but should also be trialled in PSC.

**Table 1:** Table representing the drugs prescribed in clinics, in clinical trials or with preliminary results in mice for immune-mediated disorders targeting eQTL genes

Gene	Drug	Type	Clinical indication	Application in immune - mediated diseases	eQTL effect
*BLK*	XL-228	inhibitor	cancer	-	protective
	TG100-801	inhibitor	macular degeneration	-	protective
	ilorasertib	inhibitor	cancer	-	protective
	ENMD-981693	Inhibitor	cancer	-	protective
	dasatinib	inhibitor	cancer	alleviates symptoms of RA in mouse models	protective
*CD48*	anti-CD48	inhibitor	-	alleviates symptoms of EAE in mouse models	protective
*CTLA4*	zalifrelimab	inhibitor	cancer	-	protective
	quavonlimab	inhibitor	cancer	-	protective
	erfonrilimab	inhibitor	cancer	-	protective
	cadonilimab	inhibitor	cancer	-	protective
	tremelimumab	inhibitor	cancer	-	protective
	ipilimumab	inhibitor	cancer	-	protective
	abatacept	CTLA4-mimicking	RA, JIA, UC, T1D, MS, psoriasis	phase I - IV	protective
*ERAP2*	tosedostat	inhibitor	cancer	-	predisposing
*GBA*	afegostat	stabiliser	Gaucher’s disease	-	protective
*ITGA4*	firategrast	antagonist	MS	phase II completed	predisposing
	adrilumab	inhibitor	UC and CD	phase II completed	predisposing
	natalizumab	inhibitor	CD, MS and inflammation	phase IV	predisposing
	natalizumab	inhibitor	RA	phase II terminated	predisposing
	vedolizumad	inhibitor	CD, UC and immune system disease	phase IV	predisposing
	vedolizumad	inhibitor	coeliac disease	phase II terminated	predisposing
*PTGER4*	rivenprost	agonist	UC	phase II terminated	protective
	dinoprostone	agonist	pain/pregnancy	-	protective
	CR-6086	antagonist	RA	phase II completed	protective
	grapiprant	antagonist	osteoarthtis/cancer	phase I completed	protective
*IL4R*	dupilumab	antagonist	asthma	phase III	protective
	dupilumab	antagonist	eczema	phase IV	protective
	cintredekin besudotox	binding agent	cancer	phase III	protective

*MS* multiple sclerosis, *UC* ulcerative colitis, *CD* Crohn’s disease, *RA* rheumatoid arthritis, *JIA* juvenile idiopathic arthritis, *T1D* type 1 diabetes, *EAE* experimental autoimmune encephalomyelitis.

Finally, we reasoned that if a genetic variant is associated with the protein level, this will provide further evidence for the causal role of a protein in each of the disease groups. Therefore, we colocalised protein QTLs (pQTLs)^30^ with factor-associated loci. We identified five colocalizations in F_gut_, three in F_aid_ and five in F_alrg_ with PP4 ≥ 0.9 (Supplementary Data 10). In addition, to determine whether an increase in protein level predicts increased disease risk, we used MR (Supplementary Fig. 7a, Supplementary Data 11). For example, we observed that a locus associated with F_alrg_ modulates the level of LRRC32, and an increase in LRRC32 increases the risk of F_alrg_ group of diseases (Supplementary Fig. 7b). LRRC32 regulates TGF-ß signalling and is a well-known regulator of inflammation^31^. Importantly, three out of 13 colocalizing pQTLs are known drug targets for immune-mediated diseases (IL6R, IL2RA and ERAP2) (Supplementary Fig. 7c).

Taken together, our data show that understanding the pleiotropy of genetic associations can reveal common disease mechanisms, identify novel drug targets and offer evidence for drug repurposing.

## Discussion

In this work, we used genomic SEM to investigate the common genetic factors predisposing to multiple immune-mediated diseases. We identified three broad categories of immune-mediated diseases: diseases affecting the gastrointestinal tract, rheumatic and systemic disorders, and allergic diseases. Surprisingly, underlying factors affecting the pathogenesis of each of these disease groups had a highly specific pattern of genetic associations, with only 13/301 loci being shared across these groups. This suggests that there is a genetic similarity between diseases within a group, but that the associated loci are highly distinct across groups. Importantly, as LDSC and genomic SEM control for the sample overlap in GWAS studies, disease groupings are not confounded by sharing of the samples^16,17^.

The identified groups agree with previous epidemiological findings. For example, T1D was grouped with rheumatic diseases including RA, which is in line with reports that patients with T1D but not T2D have an increased risk of RA (OR = 4.9)^32^. Similarly, ~70% of patients with PSC have IBD, with UC being the most prevalent^33^. Our study shows that there are common genetic mechanisms driving the pathogenesis of these diseases and suggests that creating cross-disorder cohorts of immune diseases could increase the power to identify causal pathogenic processes.

Importantly, over 90% of identified loci acted via common factors, rather than independently on each of the diseases. Therefore, we sought to identify transdiagnostic risk pathways to uncover biological processes whose perturbation affects each of the disease groups. Our study showed that despite associated loci being highly factor specific, they converged on perturbing the same pathways involved in T cell activation, differentiation and cytokine signalling. F_gut_ and F_aid_ and F_alrg_-associated loci were enriched in the JAK-STAT signalling pathway, although there were only three overlapping genes driving the pathway enrichment in each of these groups. Similarly, out of 53 genes that are enriched for lymphocyte activation, only 7 were shared across at least two factors. Therefore, one can speculate that perturbations at different nodes which regulate T cell activation and cytokine signalling are partially responsible for driving different disease outcomes. Recent advances in CRISPR editing in T cells and its subpopulations^34,35^ will be instrumental to elucidate the differential effects of perturbing each node within shared pathways.

Finally, it has been widely demonstrated that supporting preclinical data with genetic evidence can significantly increase the chance of developing successful drugs^36^. Therefore, understanding how trans-diagnostic variants regulate gene expression can help to identify novel drug targets or provide additional evidence for existing trials. Here we colocalized the factor-associated loci with sc-eQTL derived from the OneK1K cohort. To date, OneK1K is the largest study containing single-cell RNA sequencing (scRNA-seq) data from 982 donors and 1.27 million PMBCs. We showed that eight of these colocalizing genes are known drug targets offering further genetic support for their potential therapeutic effect. In addition, given that the assessed variants are pleiotropic, our results imply that identified drugs could be repurposed for diseases within the same group. For example, our data shows that the increase in expression of a key immune regulator *CTLA4* is protective against the F_aid_ group of diseases. The property of CTLA-4 to regulate the immune system has long been exploited in the treatment of RA^12^. Similarly, an inhibitor for Integrin Subunit Alpha 4, ITGA4 has been trialled in UC and CD (Open Targets database). Our data gives further genetic evidence that an increase in *ITGA4* expression leads to an increased risk for F_gut_ diseases, and therefore it is plausible that inhibiting *ITGA4* would be beneficial not only in CD and UC but should also be trialled in PSC. However, one limitation of this study is that we identified colocalization events for 37 out of 301 loci. This highlights the urgent need for larger cohorts, which will be better powered to detect eQTLs, as well as large-scale genetic studies in immune disease patients.

A limitation of our study is that it only focussed on GWAS performed on populations of European ancestry. This is because genomic SEM and LD score regression require the samples to be drawn from the same ancestry, as linkage disequilibrium blocks and thus LD scores are ancestry-dependent^17^. While no studies have to date validated the behaviour of genomic SEM in similar settings, we would expect that the use of the GWAS datasets originating from different ancestries may lead to spurious results. Therefore, we believe that the analysis should be conducted per ancestry rather than combining GWAS of different ancestries. As the representation of global populations in immune disease GWAS increases, follow-up studies will be required to test whether our observations are fully transferable to different ancestral groups.

In conclusion, our work underscores that three groups of immune-mediated diseases do not share similarities in their genetic predisposition, but show associated loci which converge on perturbing different nodes of a common set of pathways, including in lymphocyte activation and cytokine signalling.

## Methods

### Processing of summary statistics for LD score regression

We downloaded GWAS summary statistics from published studies on the most common autoimmune disorders: T1D^7^, RA^8^, JIA^37^, SLE^38^, CD^39^, UC^39^, AST^40^, ECZ^41^ and PSC^42^ (Supplementary Data 1). Where necessary, rsIDs were added to the summary statistics using the reference file provided in the Genomic SEM repository (https://utexas.app.box.com/s/vkd36n197m8klbaio3yzoxsee6sxo11v/file/576598996073). Where necessary, chromosomes X and Y were removed and the standard error of logistic betas was calculated based on Odds Ratio confidence intervals. Summary statistics were formatted with the *munge* function from Genomic SEM R package v.0.0.5, (with default parameters) which removes all the SNPs not present in the reference file, filters out SNP with MAF < 1% and flips the alleles according to the reference file and computes z-scores. The HapMap3 reference file is provided in the Genomic SEM repository https://utexas.app.box.com/s/vkd36n197m8klbaio3yzoxsee6sxo11v/file/805005013708.

### Estimation of genetic correlation with genomic SEM

The sum of the effective sample sizes for GWAS that was meta-analysed was calculated by retrieving the information about the cohorts from the respective publications (Supplementary Data 1). We calculated the sample prevalence for each of the cohorts using the following formula

1 $${v}_{c}={n}_{{cases}}/({n}_{{cases}}+{n}_{{controls}})$$

Next, we calculated the cohort-specific sample size as follows:

2 $${{EffN}}_{c}=4\times {v}_{c}\times (1-{v}_{c})\times ({n}_{{cases}}+{n}_{{controls}})$$

Finally, we summed the *EffN*_*c*_ of each contributing cohort to compute the sum of the effective sample size:

3 $$\sum {{EffN}}_{c}$$

Where *c* are contributing cohorts (as described at https://github.com/GenomicSEM/GenomicSEM)^43^. To estimate genetic correlation we used the *ldsc* function in Genomic SEM, using the LD reference panel provided in the Genomic SEM repository (https://utexas.app.box.com/s/vkd36n197m8klbaio3yzoxsee6sxo11v/folder/119413852418).

### Factor model specification and GWAS estimation with Genomic SEM

To assign immune-mediated diseases in groups, we used the genetic correlation ≥0.4 between disease pairs, resulting in three observed disease groups. To uncover the latent factors which represent shared variance components across diseases, we modelled the genetic variance-covariance matrix across traits using genomic SEM. We computed four confirmatory factor analyses guided by the exploratory factor analysis: a) a common factor model b) a two-factor model, where one factor was loading into CD, UC, PSC, JIA, SLE, RA and T1D while the other factor was loading into Ecz and Ast. c) A three-factor model where F1 was loading into CD, UC, PSC; F2 was loading into T1D, SLE, JIA, RA, and F3 loading into Ecz and Ast; d) A four-factor model, F1 was loading into CD and UC, F2 was loading into T1D, SLE, JIA, RA, F3 was loading into Ecz and Ast and F4 was loading into PSC and UC. The fit of the models was assessed by estimating the comparative fit index (CFI) and the standardised root mean square residual (SRMR) parameters. We used CFI > 0.95 and SRMR < 0.10 as a measure of a good fit^16^. By using the disease clustering threshold (genetic correlation 0.4) and the model fit statistics thresholds (CFI > 0.95 and SRMR < 0.10) we excluded the models with one and two factors. The four-factor model was instead excluded as it partitioned the variance of PSC, CD and UC into two separate latent factors, where UC is both in factor 1 and factor 4 reducing the interpretability of a four-factor model.

Before estimating the SNP-specific effect, we aligned the summary statistics to the reference file (https://utexas.app.box.com/s/vkd36n197m8klbaio3yzoxsee6sxo11v/file/576598996073) which is used to standardise the effect sizes and SE and format the summary statistics (i.e. remove SNPs not present in the reference files and flip the alleles to match the reference) with the *sumstats* function in Genomic SEM with default parameters. SNP-specific effects of the 4,994,803 SNPs that were shared among all the nine GWAS were estimated with the *userGWAS* function with default parameters using the weighted least squares (WLS) estimation method. To evaluate whether the calculated SNP effects were acting through our three-factor model, we performed the Q_SNP_ heterogeneity tests. The heterogeneity test returns a *χ*^2^, whose null hypothesis suggests that the SNP is acting through the specified model. Therefore, rejecting the null hypothesis means that the SNP acts through a model that is different from the specified one^16,44^.

### Loci definitions and conditional analysis

We define the boundaries of each significant genomic region by identifying all the SNPs with a *p* value lower than 1 × 10^−6^. We calculated the distance among each consecutive SNP below this threshold in the same chromosome; if two SNPs were further than 250 kb apart, then they were defined as belonging to two different genomic regions. We then considered as ‘significant’ all the genomic regions where at least one SNP had a *p* value < 5 × 10^−8^. This procedure was repeated for all GWAS. Finally, we compared genomic regions between different GWAS and merged those which overlapped, redefining the boundaries as the minimum and maximum genomic position across all overlapping genomic regions.

### Processing of summary statistics for conditional analysis and colocalization

Before running conditional analysis and colocalization, summary statistics (traits and factors) were processed with the Bioconductor MungeSumstats package^45^. We specify the parameters to the MungeSumstat function to: align the summary statistics to reference genome to the build GRCh7 (1000genomes Phase2 Reference Genome Sequence hs37d5, based on NCBI GRCh37, R package ‘BSgenome.Hsapiens.1000genomes.hs37d5’ v0.99.1), flip the alleles according to the reference file, remove the SNPs which are not in the reference file (SNP locations for Homo sapiens, dbSNP Build 144, based on GRCh37.p13, R package ‘SNPlocs.Hsapiens.dbSNP144.GRCh37’ v.0.99.20), exclude the SNPs with betas or standard errors equal to 0.

### Conditional analysis and colocalization

The genomic regions defined in the previous steps are based on genomic position, but multiple association signals may be present within each genomic region. To this end, we developed a statistical approach which first divides each GWAS-significant genomic region into its component signals and then uses colocalization across different traits to group similar association signals. First, in each genomic region for each GWAS, we performed stepwise forward conditional regression using COJO^46^. The stopping criterion was that all conditional p-values were larger than 1 × 10^−4^. This led to a set of independent SNPs using all SNPs within the genomic region boundary (±100 kb). For each SNP, a conditional dataset was produced where SNPs in the genomic region were conditioned to all identified independent SNPs apart from the target one. We then considered as true signals those with *p* value < 10^−6^ or those for which the SNP with the lowest p-value was lower than 5 × 10^−8^ in the original GWAS.

This procedure was repeated on all the traits which had a significant association in the considered genomic region. We thus obtained for each trait a set of conditional datasets covering all the SNPs in the genomic region. This procedure is similar to that used by Robinson et al.^47^ but instead of using the step-wise conditioned datasets, it uses an ‘all but one’ approach.

To understand which loci were pleiotropic between traits, we ran colocalization using coloc^27^ analysis between all pairs of loci specific for each trait. Loci which colocalized with PP4 ≥ 0.9 were grouped in a single locus. We excluded the genomic regions in the HLA locus (chromosome 6—25,000,000–35,000,000) from this analysis.

### Colocalization with eQTL and pQTL data

We downloaded eQTLs from the OneK1K cohort^22^. pQTL results were obtained from DECODE genetics^30^. For each genomic region, we first identified if cis-eQTLs or cis-pQTL were present. For each identified eQTL we performed the decomposition of the locus as described above and the identified loci were colocalized with factor-associated GWAS signals. For pQTL, we did not perform the conditional analysis prior to colocalization as we did not have a reference LD panel for the Icelandic population. Attempts of using a different LD reference set resulted in hundreds of putatively independent loci, which are likely false positives. Therefore we tested only the single main effect. To identify a colocalizing signal we required PP4 ≥ 0.9. To identify the direction of the effect of the increase in gene expression for the colocalizing loci, we used Mendelian Randomization using the Wald ratio method (TwoSampleMR R package^48^). We used the SNP with the smallest p-value in the conditional analysis as an instrument variable. Significant MR results (*p* value lower than 0.05) were reported. This procedure was performed per cell type.

### Cell type enrichment

To identify cell types underlying identified factors we used CELL-type Expression-specific integration for Complex Traits (CELLECT). CELLECT quantifies the association between GWAS signal and gene expression specificity using well-established models for GWAS enrichment MAGMA^20^ and S-LDSC^49^.

### Gene-based enrichment

Candidate genes were retrieved by interrogating the Variant-to-Gene (V2G) pipeline in the Open Targets Platform^19^ for the lead SNPs within the conditionally independent loci. To calculate a prioritisation score for candidate genes, the V2G pipeline takes into account molecular phenotypes (eQTL, pQTL), chromatin interactions, functional predictions and distance to the transcription start site. To identify enrichment in KEGG pathways and GO terms we used the R package gprofiler2 (v0.2.1)^50^, with default parameters. Pathway was considered significant if p-adj < 0.05. We used the R package pathview (v1.34.0)^51^ to represent the KEGG pathways and to highlight factor-specific genes. The diagram shown in Fig. 3b was created with biorender.com using the KEGG pathway as a reference.

### Identification of drug targets

Open Targets Platform^29^ (v.22.06) was used to identify drug targets for eQTL genes. This website was queried on (29th August 2022).

### Reporting summary

Further information on research design is available in the Nature Portfolio Reporting Summary linked to this article.

## Supplementary information

Supplementary information

Description of Additional Supplementary Files

Supplementary Data 1

Supplementary Data 2

Supplementary Data 3

Supplementary Data 4

Supplementary Data 5

Supplementary Data 6

Supplementary Data 7

Supplementary Data 8

Supplementary Data 9

Supplementary Data 10

Supplementary Data 11

Reporting Summary

## Data Availability

Publicly available GWAS summary statistics were downloaded from the GWAS catalogue or provided by the authors of the respective publications. MAF reference file, HapMap3 reference file and LD reference panel are provided in the Genomic SEM repository. Gut immune cell atlas: https://cellgeni.cog.sanger.ac.uk/gutcellatlas/Full_obj_log_counts_soupx_v2.h5ad. Lung immune cells scRNA-seq data: https://covid19.cog.sanger.ac.uk/madissoon19_lung.processed.h5ad.

## Code availability

All codes are available at https://github.com/SoskicLab/aid_sharing.
