# Supplementary material for: Cross-disorder genetic analysis of immune diseases reveals distinct gene associations that converge on common pathways
Source: Nat Commun. 2023 May 12;14:2743. doi: 10.1038/s41467-023-38389-6 (PMC10182075; doi:10.1038/s41467-023-38389-6)
Supplement: Supplementary file 1 — Supplementary information [file 41467_2023_38389_MOESM1_ESM.pdf]

Cross-disorder genetic analysis of immune diseases reveals distinct gene associations that converge on common pathways

Supplementary Figures

a)

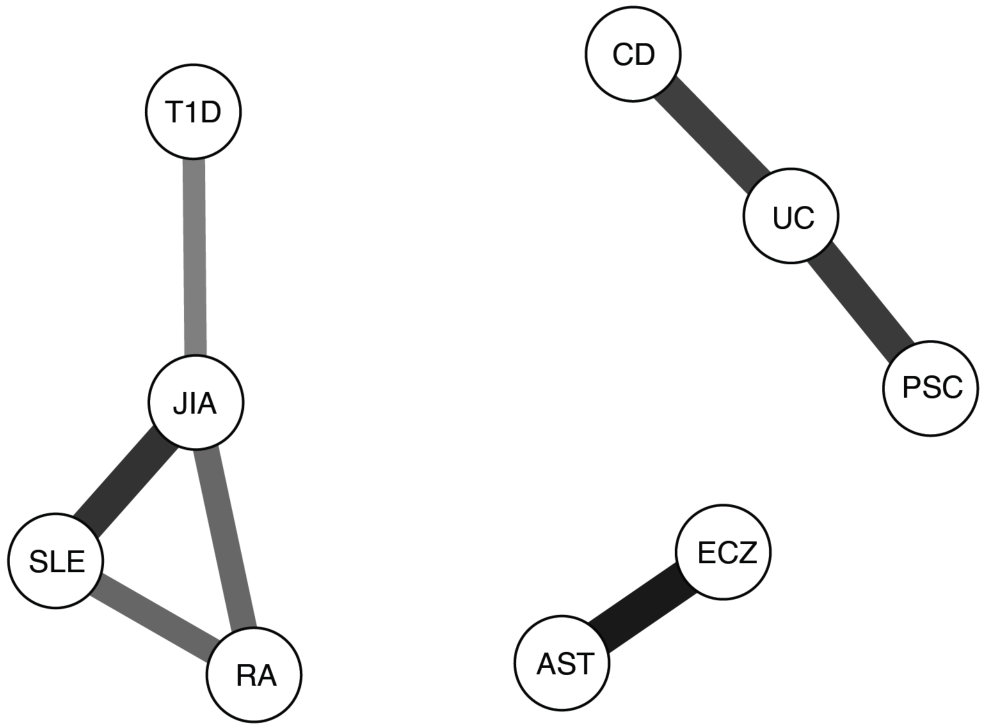

Supplementary Figure 1. The network plot of LDSC genetic correlations among nine autoimmune diseases. a) The grey scale and edge width represent the genetic correlation. Genetic correlations ≥ 0.4 are shown.

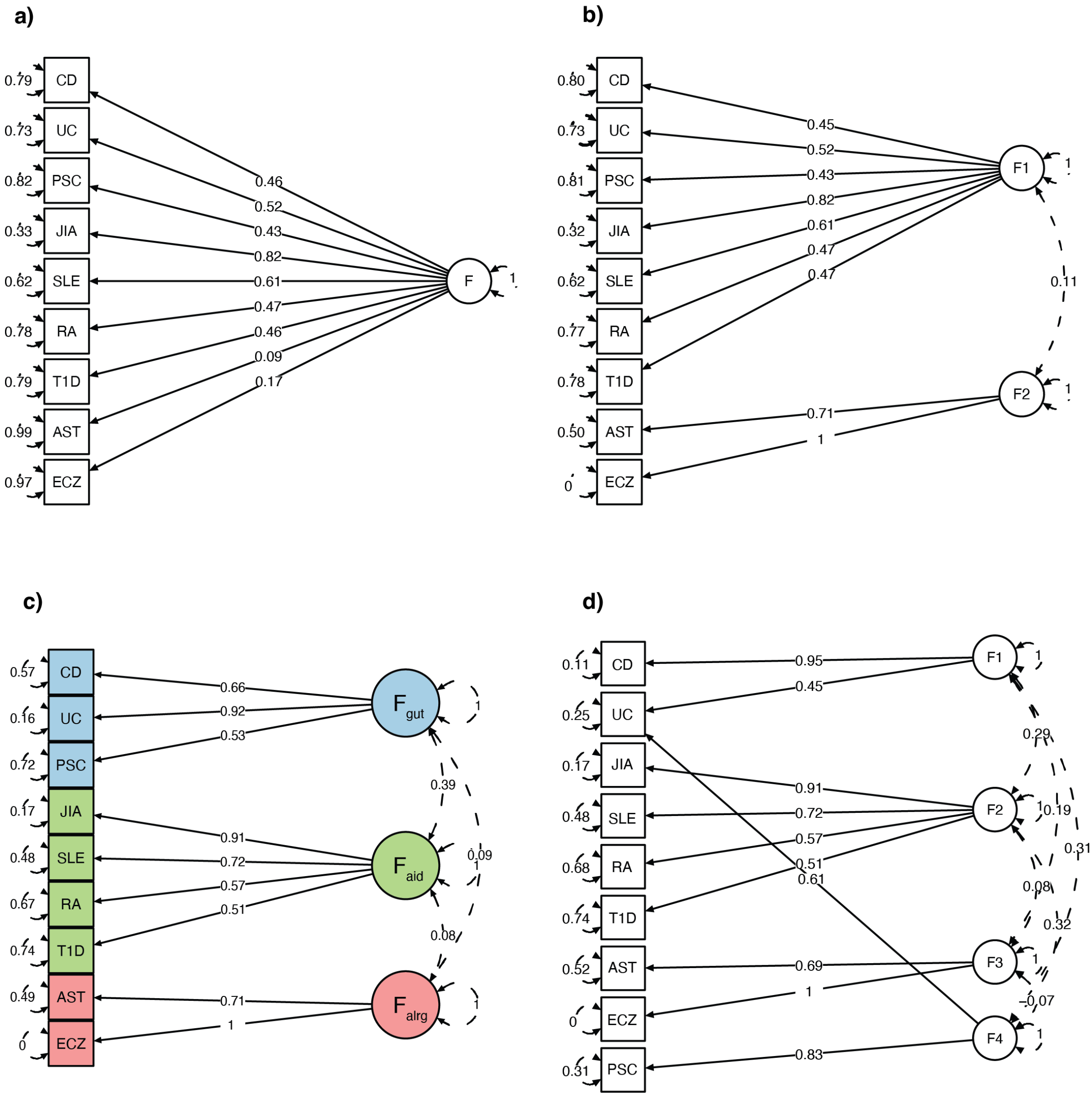

e)

Model (number of factors)	χ^2	df	P-value χ^2	AIC	CFI	SRMR
1	493.24	27	3.8E-87	529.24	0.58	0.16
2	288.02	26	5.2E-46	326.02	0.76	0.11
3	61.26	24	4.2E-05	103.26	0.97	0.06
4	32.67	20	3.7E-02	82.67	0.99	0.04

Supplementary Figure 2. Genomic SEM model statistics. a-d) We tested four genomic SEM models. Standardised loadings (one-headed arrows), residual variances (two-headed arrows connecting the variable with itself) and covariances (two-headed arrows connecting latent variables) are shown. **e)** Table of factor analysis fit statistics for each of the four models. df, degrees of freedom of the model. P-values were calculated from a χ^2 distribution with the reported degrees of freedom (df); AIC, Akaike Information Criterion; CFI, Comparative Fit Index; SRM, Standardised Root Mean Square Residual.

a)

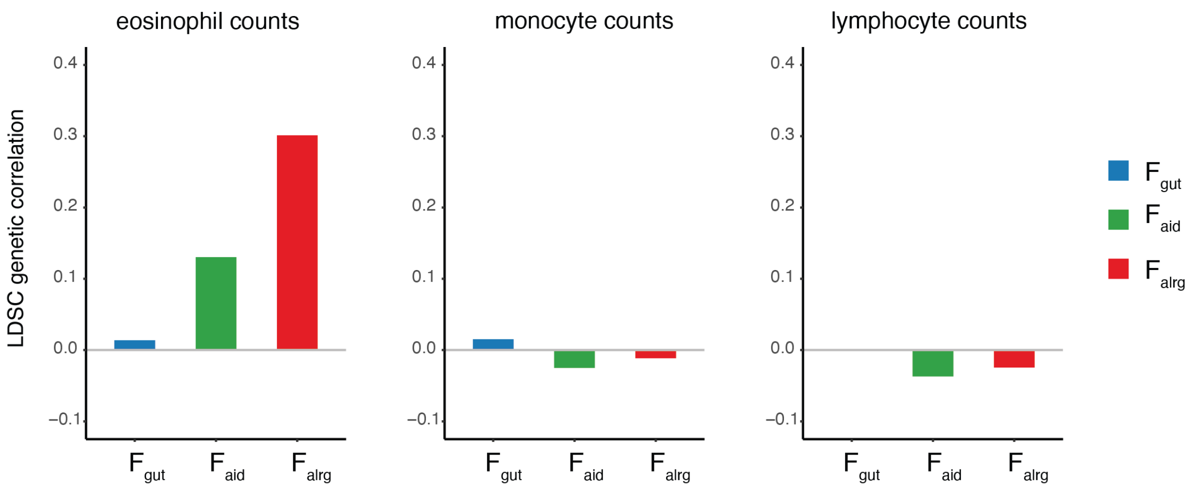

Supplementary Figure 3. Eosinophil counts are correlated with F_{alrg}. a) LDSC genetic correlations between factors and blood cell counts. Blue, green and red represent F_{gut}, F_{aid} and F_{alrg} respectively.

a)

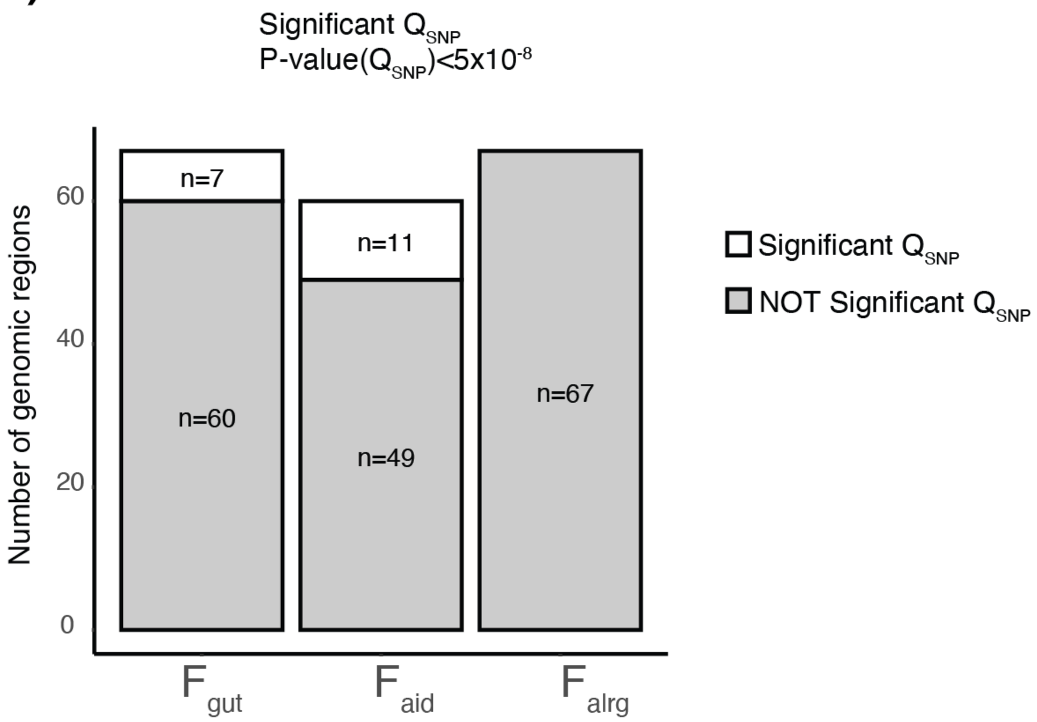

Supplementary Figure 4. Q_{SNP} statistics of factor associated loci. a) The bar plot shows the number of lead SNPs which had a significant Q_{SNP} (in white) and not significant (in grey). Genomic SEM (WLS estimation method) was used to conduct the main GWAS and to estimate the independent model for Q_{SNP} calculation. P-values of Q_{SNP} were calculated from a χ^2 distribution with 2, 3 and 1 degrees of freedom for F_{gut}, F_{aid} and F_{alrg} respectively.

a)

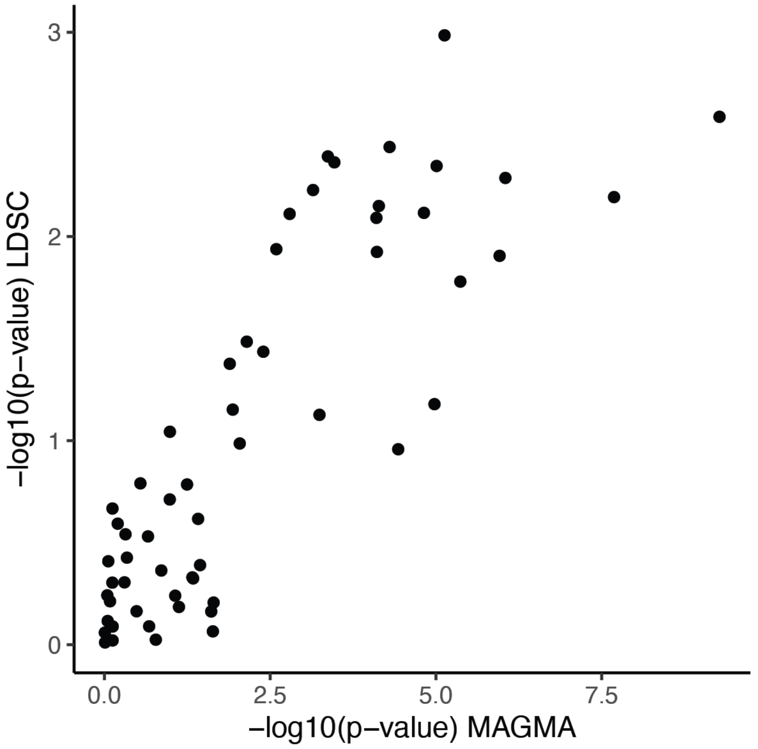

Supplementary Figure 5. Comparison of LDSC and MAGMA enrichments. a) Dot plot shows correlation of -log_{10}(p-value) between MAGMA and LDSC outputs for OneK1K cohort**.** P-values were estimated with MAGMA and LDSC (one-sided test).

a)

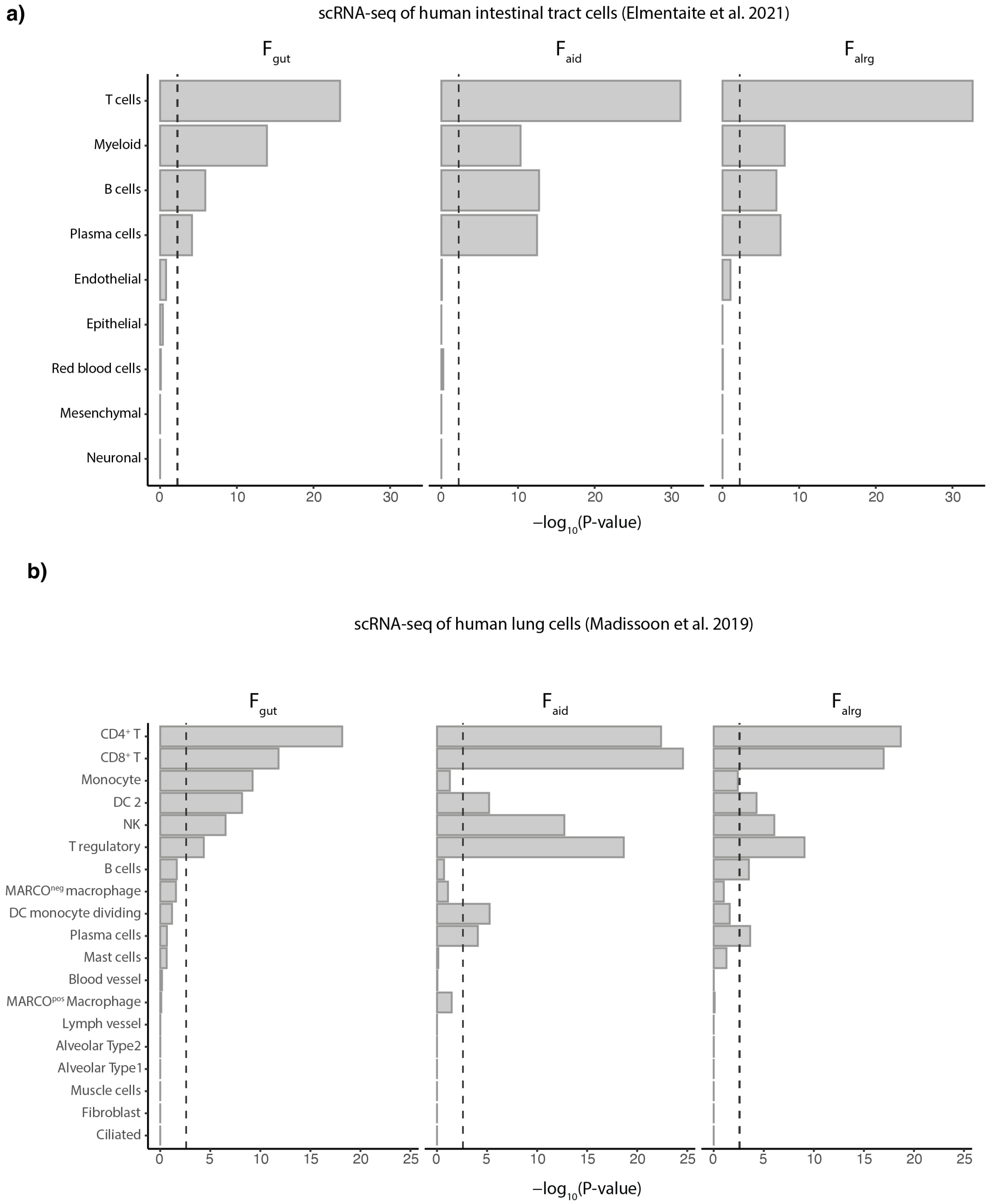

Supplementary Figure 6. Factor-associated loci are specifically enriched in tissue immune cells. a-b) MAGMA gene-property results of intestinal cells(**a**) and lung cells(**b**). The barplot shows -$\log_{10}$(p-value) of the enrichment. P-values were estimated with MAGMA for testing the null-hypothesis (one-sided test) that the risk variants were not enriched in the cell-types.

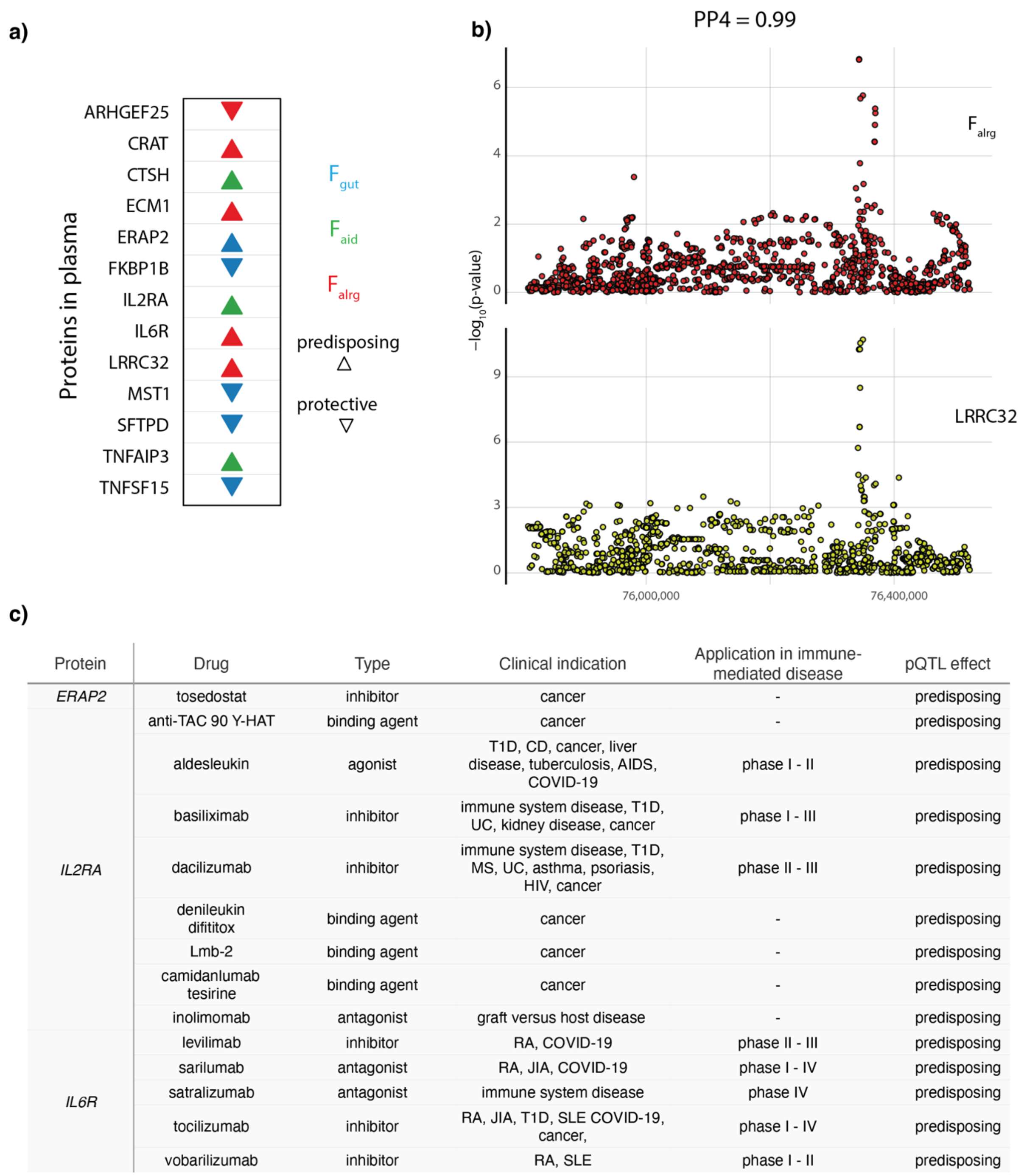

c)

Protein	Drug	Type	Clinical indication	Application in immune-mediated disease	pQTL effect
ERAP2	tosedostat	inhibitor	cancer	-	predisposing
IL2RA	anti-TAC 90 Y-HAT	binding agent	cancer	-	predisposing
	aldesleukin	agonist	T1D, CD, cancer, liver disease, tuberculosis, AIDS, COVID-19	phase I - II	predisposing
	basiliximab	inhibitor	immune system disease, T1D, UC, kidney disease, cancer	phase I - III	predisposing
	dacilizumab	inhibitor	immune system disease, T1D, MS, UC, asthma, psoriasis, HIV, cancer	phase II - III	predisposing
	denileukin difititox	binding agent	cancer	-	predisposing
	Lmb-2	binding agent	cancer	-	predisposing
	camidanlumab tesirine	binding agent	cancer	-	predisposing
	inolimomab	antagonist	graft versus host disease	-	predisposing
IL6R	levilimab	inhibitor	RA, COVID-19	phase II - III	predisposing
	sarilumab	antagonist	RA, JIA, COVID-19	phase I - IV	predisposing
	satralizumab	antagonist	immune system disease	phase IV	predisposing
	tocilizumab	inhibitor	RA, JIA, T1D, SLE COVID-19, cancer,	phase I - IV	predisposing
	vobarilizumab	inhibitor	RA, SLE	phase I - II	predisposing

Supplementary Figure 7. Colocalization of protein QTLs. a) Triangles pointing upwards indicate that an increase in protein level increases disease risk, while triangles point downwards indicate decrease of disease risk. Blue, green and red represent F_{gut}, F_{aid} and F_{alrg} respectively. Mendelian Randomization with Wald ratio method was used to estimate the p-value (two-sided). Only significant MR results (p-value <0.05) are shown. **b)** Locus-zoom plot representing the colocalization between the level of LRRC2 protein in plasma and F_{alrg}. P-values refer to the SNP p-values derived from the F_{alrg} GWAS and from the p-QTL dataset. **c)** Table representing pQTLs which are known drug targets. MS, multiple sclerosis; UC, ulcerative colitis; CD, Crohn's disease; RA, rheumatoid arthritis; JIA, juvenile idiopathic arthritis; T1D, type 1 diabetes; SLE, systemic lupus erythematosus.
