# Supplementary material for: Cross-disorder genetic analysis of immune diseases reveals distinct gene associations that converge on common pathways
Source: Nat Commun. 2023 May 12;14:2743. doi: 10.1038/s41467-023-38389-6 (PMC10182075; doi:10.1038/s41467-023-38389-6)
Supplement: Supplementary file 2 — Description of Additional Supplementary Files [file 41467_2023_38389_MOESM2_ESM.pdf]

Description of Additional Supplementary Files

File Name: Supplementary Data 1
Description: **GWAS studies used in the analysis.**

File Name: Supplementary Data 2
Description: **LD score regression results.** Genetic correlation p-values (two-sided) from LD score regression genetic correlation analysis. Standard errors of LD score regression genetic correlations. LDscore regression covariance matrix (on the liability scale).

File Name: Supplementary Data 3
Description: **Significant genomic regions in latent factors and traits GWAS.** Genomic SEM (WLS estimation method) was used to conduct the GWAS.

File Name: Supplementary Data 4
Description: **Conditionally independent loci.**

File Name: Supplementary Data 5
Description: **Colocalization of loci among factors and immune diseases GWAS.**

File Name: Supplementary Data 6
Description: **Significant KEGG pathways enriched in latent factors.** P-values were calculated with the hypergeometric test and corrected for multiple testing with the gprofiler-g:SCS method.

File Name: Supplementary Data 7
Description: **Significant GO terms enriched in latent factors.** P-values were calculated with the hypergeometric test and corrected for multiple testing with the gprofiler-g:SCS method.

File Name: Supplementary Data 8
Description: **Colocalization results between immune latent factors and eQTLs.**

File Name: Supplementary Data 9
Description: **Mendelian Randomization Results of colocalizing eQTL.** Mendelian Randomization with the Wald ratio method was used to estimate the p-values (two-sided).

File Name: Supplementary Data 10
Description: **Colocalisation results between immune latent factors and pQTL.**

File Name: Supplementary Data 11
Description: **Mendelian Randomization results of colocalizing pQTL.** Mendelian Randomization with Wald ratio method was used to estimate the p-values (two-sided).
