# Supplementary material for: Cross-disorder genetic analysis of immune diseases reveals distinct gene associations that converge on common pathways
Source: Nat Commun. 2023 May 12;14:2743. doi: 10.1038/s41467-023-38389-6 (PMC10182075; doi:10.1038/s41467-023-38389-6)
Supplement: Supplementary file 14 — Reporting Summary [file 41467_2023_38389_MOESM14_ESM.pdf]

nature portfolio

Corresponding author(s): Blagoje Soskic

Last updated by author(s): Apr 18, 2023

Reporting Summary

Nature Portfolio wishes to improve the reproducibility of the work that we publish. This form provides structure for consistency and transparency in reporting. For further information on Nature Portfolio policies, see our Editorial Policies and the Editorial Policy Checklist.

Statistics

For all statistical analyses, confirm that the following items are present in the figure legend, table legend, main text, or Methods section.

n/a	Confirmed	
☒	☐	The exact sample size (*n*) for each experimental group/condition, given as a discrete number and unit of measurement
☐	☒	A statement on whether measurements were taken from distinct samples or whether the same sample was measured repeatedly
☐	☒	The statistical test(s) used AND whether they are one- or two-sided<br>*Only common tests should be described solely by name; describe more complex techniques in the Methods section.*
☐	☒	A description of all covariates tested
☐	☒	A description of any assumptions or corrections, such as tests of normality and adjustment for multiple comparisons
☐	☒	A full description of the statistical parameters including central tendency (e.g. means) or other basic estimates (e.g. regression coefficient) AND variation (e.g. standard deviation) or associated estimates of uncertainty (e.g. confidence intervals)
☐	☒	For null hypothesis testing, the test statistic (e.g. *F*, *t*, *r*) with confidence intervals, effect sizes, degrees of freedom and *P* value noted<br>*Give P values as exact values whenever suitable.*
☐	☒	For Bayesian analysis, information on the choice of priors and Markov chain Monte Carlo settings
☐	☒	For hierarchical and complex designs, identification of the appropriate level for tests and full reporting of outcomes
☒	☐	Estimates of effect sizes (e.g. Cohen's *d*, Pearson's *r*), indicating how they were calculated

Our web collection on statistics for biologists contains articles on many of the points above.

Software and code

Policy information about availability of computer code

Data collection	No software was used for data collection.
Data analysis	All the analyses was conducted in R (v4.1.0). genomicSEM (v0.0.5) was used to munge the summary statistics and run multivariate LDscore regression using the reference files provided by the package authors. SNP-specific effects were estimated with the userGWAS function with default parameters using the weighted least squares (WLS) estimation method, MAF filter > 0.05. Summary statistics of immune factors and immune diseases were formated with the MungeSumstat package (v1.2.4). Summary statistics were aligned to the reference genome (1000genomes Phase2 Reference Genome Sequence hs37d5, based on NCBI GRCh37, R package 'BSgenome.Hsapiens.1000genomes.hs37d5' v0.99.1). Alleles were aligned to the reference file (SNP locations for Homo sapiens, dbSNP Build 144, based on GRCh37.p13, R package 'SNPlocs.Hsapiens.dbSNP144.GRCh37' v.0.99.20). Conditional analysis was conducted using COJO in the GCTCA software (v. gcta64 1.94.0beta). Colocalisation analysis was performed using coloc (v.5.1.4) and Mendelian randomization was conducted using the R package TwoSampleMR (v0.5.6). The Open Targets Platform (v22.11) was queried for retrieving candidate genes and drug targets. Pathway enrichment analysis was performed with gprofiler2 (v0.2.1).Cell type enrichment was performed with the MAGMA implementation in the CELLECT framework (v1.3.0).

For manuscripts utilizing custom algorithms or software that are central to the research but not yet described in published literature, software must be made available to editors and reviewers. We strongly encourage code deposition in a community repository (e.g. GitHub). See the Nature Portfolio guidelines for submitting code & software for further information.

Data

Policy information about availability of data

All manuscripts must include a data availability statement. This statement should provide the following information, where applicable:

- Accession codes, unique identifiers, or web links for publicly available datasets
- A description of any restrictions on data availability
- For clinical datasets or third party data, please ensure that the statement adheres to our policy

Publicly available GWAS summary statistics were downloaded from GWAS catalog or provided by the authors of the respective publications.
Crohn's disease (CD) http://ftp.ebi.ac.uk/pub/databases/gwas/summary_statistics/GCST004001-GCST005000/GCST004132/cd_build37_40266_20161107.txt.gz,
Ulcerative colitis (UC) http://ftp.ebi.ac.uk/pub/databases/gwas/summary_statistics/GCST004001-GCST005000/GCST004133/uc_build37_45975_20161107.txt.gz,
Rheumatoid arthritis (RA) summary statistics were obtained from Prof Yukinori Okada,
Asthma (Ast) http://ftp.ebi.ac.uk/pub/databases/gwas/summary_statistics/GCST010001-GCST011000/GCST010042/HanY_prePMID_asthma_UKBB.txt.gz,
Systemic lupus erythematosus (SLE) http://ftp.ebi.ac.uk/pub/databases/gwas/summary_statistics/GCST003001- GCST004000/GCST003156/bentham_2015_26502338_sle_efo0002690_1_gwas.sumstats.tsv.gz,
Primary sclerosing cholangitis (PSC) http://ftp.ebi.ac.uk/pub/databases/gwas/summary_statistics/GCST004001-GCST005000/GCST004030/ipscsg2016.result.combined.full.with_header.txt,
Juvenile idiopathic arthritis (JIA) http://ftp.ebi.ac.uk/pub/databases/gwas/summary_statistics/GCST90010001-GCST90011000/GCST90010715/GCST90010715_buildGRCh37.tsv,
Type 1 diabetes (T1D) http://ftp.ebi.ac.uk/pub/databases/gwas/summary_statistics/GCST90014001-GCST90015000/GCST90014023/GCST90014023_buildGRCh38.tsv,
Atopic dermatitis/ eczema (Ecz) http://ftp.ebi.ac.uk/pub/databases/gwas/summary_statistics/GCST90027001-GCST90028000/GCST90027161/GCST90027161_buildGRCh38.tsv.gz,
Allergies http://ftp.ebi.ac.uk/pub/databases/gwas/summary_statistics/GCST005001-GCST006000/GCST005038/SHARE-without23andMe.LDSCORE-GC.SE-META.v0.gz,
Lymphocyte counts http://ftp.ebi.ac.uk/pub/databases/gwas/summary_statistics/GCST90002001-GCST90003000/GCST90002388/GCST90002388_buildGRCh37.tsv,
Monoyte counts http://ftp.ebi.ac.uk/pub/databases/gwas/summary_statistics/GCST90002001-GCST90003000/GCST90002393/GCST90002393_buildGRCh37.tsv,
Eosinophil counts, http://ftp.ebi.ac.uk/pub/databases/gwas/summary_statistics/GCST90002001-GCST90003000/GCST90002381/GCST90002381_buildGRCh37.tsv

MAF reference file is provided in the Genomic SEM repository https://utexas.app.box.com/s/vkd36n197m8klbaio3yzoxsee6sxo11v/file/576598996073

HapMap3 reference file is provided in the Genomic SEM repository https://utexas.app.box.com/s/vkd36n197m8klbaio3yzoxsee6sxo11v/file/805005013708

LD reference panel is provided in the Genomic SEM repository
https://utexas.app.box.com/s/vkd36n197m8klbaio3yzoxsee6sxo11v/folder/119413852418

Gut immune cells scRNA-seq data
https://cellgeni.cog.sanger.ac.uk/gutcellatlas/Full_obj_log_counts_soupx_v2.h5ad

Lung immune cells scRNA-seq data
https://covid19.cog.sanger.ac.uk/madissoon19_lung.processed.h5ad

Human research participants

Policy information about studies involving human research participants and Sex and Gender in Research.

Reporting on sex and gender	NA
Population characteristics	NA
Recruitment	NA, publicly available datasets were used.
Ethics oversight	NA, publicly available datasets were used.

Note that full information on the approval of the study protocol must also be provided in the manuscript.

Field-specific reporting

Please select the one below that is the best fit for your research. If you are not sure, read the appropriate sections before making your selection.

☒ Life sciences ☐ Behavioural & social sciences ☐ Ecological, evolutionary & environmental sciences

For a reference copy of the document with all sections, see nature.com/documents/nr-reporting-summary-flat.pdf

Life sciences study design

All studies must disclose on these points even when the disclosure is negative.

Sample size	We collected GWAS summary statistics from European populations, and we selected studies that used genome-wide genotyping arrays with a sample size >5000, as it is required for accurate estimation of LD score regression.
Data exclusions	We excluded SNPs that were not shared among nine immune-mediated diseases. Due to challenges of the HLA region (i.e. the LD structure) we also excluded the genomic regions in the HLA locus (chromosome 6 - 25,000,000-35,000,000, build37) from this analysis. As noted previously, we used datasets from European populations only as LD score regression requires cohorts to be within the same ethnic group.
Replication	Replication was not applicable as we downloaded publicly available summary statistics.
Randomization	Randomization was not applicable as we downloaded publicly available summary statistics.
Blinding	Blinding was not applicable as we downloaded publicly available summary statistics.

Reporting for specific materials, systems and methods

We require information from authors about some types of materials, experimental systems and methods used in many studies. Here, indicate whether each material, system or method listed is relevant to your study. If you are not sure if a list item applies to your research, read the appropriate section before selecting a response.

Materials & experimental systems

n/a	Involved in the study	
☒	☐	Antibodies
☒	☐	Eukaryotic cell lines
☒	☐	Palaeontology and archaeology
☒	☐	Animals and other organisms
☒	☐	Clinical data
☒	☐	Dual use research of concern

Methods

n/a	Involved in the study	
☒	☐	ChIP-seq
☒	☐	Flow cytometry
☒	☐	MRI-based neuroimaging
